# Corridor quality affects net movement, size of dispersers, and population growth in experimental microcosms

**DOI:** 10.1007/s00442-020-04834-2

**Published:** 2021-01-09

**Authors:** Dongbo Li, Christopher F. Clements, Isobel L. G. Shan, Jane Memmott

**Affiliations:** School of Biological Sciences, University of Bristol, Life Sciences Building, 24 Tyndall Avenue, Bristol, BS8 1TQ UK

**Keywords:** *Folsomia candida*, Inter-patch distance, Habitat fragmentation, Movement rates, Metapopulation

## Abstract

Corridors are expected to increase species dispersal in fragmented habitats. However, it remains unclear how the quality of corridors influences the dispersal process, and how it interacts with corridor length and width. Here we investigate these factors using a small-scale laboratory system where we track the dispersal of the model organism Collembola *Folsomia candida*. Using this system, we study the effects of corridor length, width, and quality on the probability of dispersal, net movement, body size of dispersers, and the rate of change in population size after colonization. We show that corridor quality positively affected dispersal probability, net movement, and the rate of change in population size in colonised patches. Moreover, corridor quality significantly affected the size of dispersers, with only larger individuals dispersing through poor quality corridors. The length and width of corridors affected both the rate at which populations increased in colonised patches and the net number of individuals which dispersed, suggesting that these physical properties may be important in maintaining the flow of individuals in space. Our results thus suggest that corridor quality can have an important role in determining not only the probability of dispersal occurs but also the phenotypes of the individuals which disperse, with concomitant effects on the net movement of individuals and the rate of change in population size in the colonised patches.

**Supplementary Information:**

The online version contains supplementary material available at 10.1007/s00442-020-04834-2.

## Introduction

Corridors in fragmented landscapes have been regarded as one of the key approaches for mitigating the negative effects of habitat fragmentation. It has been shown that wildlife corridors promote the dispersal of plants and animals (Gilbert-Norton et al. 2010), rescue populations from extinction (Rantalainen et al. 2006), and maintain species richness at both small (Gilbert et al. 1998) and large scales (Damschen et al. 2006). Although the effectiveness of corridors remains controversial (Beier and Noss 1998; Gilbert-Norton et al. 2010), the potential for corridors to positively impact biodiversity in a fragmented world has led to them becoming a widely used tool for biodiversity conservation and wildlife management (Chetkiewicz et al. 2006; Haddad et al. 2014).

The role of corridors in promoting dispersal is likely to be particularly important for small and isolated populations, which have increased the risks of inbreeding depression and genetic drift (Hedrick and Kalinowski 2000). Isolated populations can be rescued by the presence of corridors (Gilbert et al. 1998), enhancing the functional connectivity and population viability in fragmented landscapes. This “corridor effect”, however, will be influenced by corridor features and the effects of the physical properties of corridors such as length and width have been demonstrated in previous studies. For example, in a large-scale experiment, Haddad (1999) found that there were more frequent inter-patch movements in shorter corridors. By simulating individual movements, Tischendorf and Wissel (1997) showed that the probability of moving in corridors for small animals increases with corridor width. However, corridor length and width do not always have effects on dispersal or patch colonisation and, some studies report no significant corridor length and width effects (e.g. Darcy and Eggleston 2005; Öckinger and Smith 2008; Ives et al. 2011). More importantly, the ratio of corridor length to width can determine whether species use corridors for movement (Sieving et al. 2000)—a key concept that controls the trade-off of between construction cost and conservation needs (Pouzols and Moilanen 2014). While much less studied, corridor quality is another factor that affects corridor effectiveness (Bennett et al. 1994; Rantalainen et al. 2005). For example, by modelling the demographics of the white-footed mouse *Persomyscus leucopus* metapopulation, Henein and Merriam (1990) found that corridor quality has positive effects on the size of the population. Moreover, if the corridor quality is poor, mortality can occur during dispersal in corridors (Christie and Knowles 2015). However, to date, very little is known about how corridor quality effects the rate of dispersal of species.

Dispersal ability is affected not only by corridor features but also by the traits of the dispersing species, with effects on the source and colonising populations (Dewhirst and Lutscher 2009; De Bie et al. 2012). For example, young males are found to be better dispersers than others in soil mite populations (Bowler and Benton 2009b), therefore the patch sex ratio and age structure can be affected according to their different dispersal abilities even under same corridor conditions. Closely linked with individual fitness and behaviour, body size has proved to be a predictor of dispersal distance for many species. In laboratory experiments, Johnson and Wellington (1983) found that the distance dispersed by the soil dwelling Collembolan *Folsomia Candida* (Willem) was a linear function of body size, as large individuals showed a tendency to disperse to more distant areas. By studying 77 bird and 88 mammal species, Sutherland et al. (2000) also found that larger species can disperse farther than smaller ones. In summary, the effect of corridors on dispersal populations can be complicated, as the corridor’s features not only regulate how they disperse in the corridor but can also control which of them disperse too. If corridors are too long or the quality poor, only a few highly mobile and large body-sized individuals are likely to colonise habitat patches, leading to changes in population size structure which may be destabilising (Filin and Ovadia 2007; Ozgul et al. 2012). For example, Ohlberger et al. (2020) found that a narrow distribution of body size in *Esox lucius* offspring population is more likely to drive a lower population stability.

While large scale manipulative field experiments are likely to remain the gold standard for testing the effectiveness of corridors (e.g. Ewers et al. 2011; Damschen et al. 2019), they are rare and likely to remain so because of the logistical challenges of running experiments at this scale. Small scale experimental systems offer an alternative approach which can provide useful arenas for testing landscape-level ecological process which are otherwise hard to manipulate and observe (Srivastava et al. 2004; Benton et al. 2007; Altermatt et al. 2015). Such systems have previously demonstrated the effects of corridors on species richness (Gilbert et al. 1998; Hoyle and Gilbert 2004), on dispersal (Bowler and Benton 2009a), and the impact of network modularity on the response to perturbations (Gilarranz et al. 2017).

Here we use a small-scale experimental system hosting the soil Collembola *Folsomia candida* to study the effects of corridors on dispersal. We constructed experimental arenas consisting of two patches connected by corridors of varying lengths and widths. In addition, we manipulated the quality of the corridor by altering the substrate—and thereby moisture levels in the corridor—a key abiotic factor for Collembola which need a humid environment to thrive. Using this system, we test how corridor length, width, and quality influence the probability of dispersal, the net movement of individuals (defined as the number of individuals which have dispersed through the corridor), which individuals disperse (large or small ones), and how this dispersal influences the rate of change in population size in the colonised patches. We predict that: (1) good quality corridors increase the probability of dispersal and increase net movement of individuals, and consequently the rate at which populations in the newly invaded patches increase; (2) increasing corridor length will decrease the net movement, as individuals have to travel further through inhospitable territory; (3) increasing corridor width will increase net movement, as more individuals are likely to move to the second patch; (4) individuals with larger body sizes are more likely to disperse through poor quality corridors as they are more robust to inhospitable conditions.

## Materials and methods

### Study organism

Our model study species is the soil Collembola, *Folsomia candida* (Collembola, Isotomidae). *F. candida* was reared at room temperature (c. 19 ℃), in high humidity conditions with dry yeast for food prior to the experiment. The growth and fecundity of *F. candida* cultures are influenced by environmental factors such as food resources and temperatures (Fountain and Hopkin 2005; Hafer et al. 2011). At 20 ℃, the eggs take 7–10 days to hatch and then take 21–24 days to reach sexual maturity (Fountain and Hopkin 2005). As a soil-dwelling arthropod, *F. candida* species prefers dark habitats rather than light habitats (Ruiz et al. 2017). Humidity is essential to *F. candida*, although they can survive in relatively dry soil conditions (Sjursen et al. 2001; Hilligsøe and Holmstrup 2003).

### Experimental design

The experimental arenas were made from 3D printed plastic and consisted of two habitat patches (circular: 5 cm in diameter and 1.5 cm in height) connected by a single corridor. A base layer of Plaster of Paris, 0.5 cm thick, was added to each habitat patch and 0.5 ml water was added twice a week to maintain a humid environment. The Plaster of Paris was dyed black to facilitate the counting of the white Collembola.

Corridor length, width, and quality were varied in a fully factorial experiment as follows. Corridors were either short (7 cm) or long (14 cm), narrow (0.5 cm) or wide (1 cm), or good or poor quality. Corridor quality was varied by adding either adding Plaster of Paris (as used in the habitat patches to maintain humidity) to the corridor (good quality) or leaving the plastic corridor base uncovered (poor quality). Each combination of corridor length, width, and quality was replicated 10 times, leading to a total of 80 microcosms.

On day 0 of the experiment the Collembola were added to one of the two habitat patches in a microcosm (henceforth referred to as the “source” patch, whilst the connected vacant patch is referred to forthwith as the “colonised” patch). Collembola are not easy to handle individually as they are very delicate, therefore, Collembola were added to microcosms by tipping a small number (mean ± SD = 57.5 ± 43) into the source patches from the cultures and then counting them afterwards to determine inoculum size (Gilarranz et al. 2017). After the inoculation, food was provided in both habitat patches once a week by adding 0.5 ml of nutritional yeast mixed into the water at a concentration of 0.006 g/l. To prevent the microcosms from drying out, a Perspex lid was clipped to the microcosms and only removed for counting. For the duration of the experiment, the microcosms were kept in a dark environment.

### Population sampling

Pilot work showed that the dispersal between habitat patches in the microcosm was likely to occur during the first few days of the experiment and therefore the Collembola were photographed more frequently at the start of the experiment. Thus, sampling took place twice a day (9:00 am and 3:00 pm) for the first five days, then once a day (9:00 am) until day 12, and subsequently twice a week (at 9:00 am) until day 21. Collembola were counted using photographs of the source and colonised patches. A camera was mounted on a tripod at a constant height and a LED ring-flash was used for supplementary lighting. The mesocosms were photographed in the same order on each occasion. We used an automated image analysis system in ImageJ/Fiji to count the numbers of individuals and measure Collembola body size in the pictures (Mallard et al. 2013). Counting is done by taking three photographs in rapid succession and comparing them to determine which *F. candida* individuals have moved in the intervening times, an approach which means that only live individuals are counted. Body size was also measured as it is a phenotypic trait linked to reproduction, survival, and dispersal probability in many organisms (Berger 2012; De Bie et al. 2012; Parsons and Joern 2014). Collembola body size was defined as the longest distance between two points of an individual, and due to limitation in the image quality, only individuals larger than 50 pixels (0.26 mm) in length were counted.

### Data analysis

We assessed how corridor length, width, and quality affect the number and identity of Collembola dispersal in three ways:

1. We used a binomial generalized linear mixed effect model (GLMM) to investigate how the different experimental treatments affect the probability that an individual dispersed through the corridors. Colonisation was recorded as a single event having occurred when the first individuals were observed in the colonised patches. We fitted a GLMM with a binomial distribution to these data, with time measured as the hours since the start of the experiment and the experimental treatments (corridor length, width, and quality) included as fixed effects. To determine whether there was an influence of experimental treatments on the time it took individuals to disperse, we included the two-way interactions between time (i.e. hours since the start of the experiment) and the experimental treatments (i.e. corridor length, width, and quality). The microcosm identity was included as a random factor to account for the variation in starting densities in the source patches among microcosms.
2. We assessed the effects of corridor length, width, and quality on the body size of the first dispersers into the colonised patches. A generalized linear mixed effect model (GLMM) with Gaussian distribution was used to test for the effects of corridor length, width, and quality, and their interactions, on the mean body size of first dispersers. The microcosm identity was included as a random effect to account for the variation in starting densities in the source patches among microcosms. Differences in the body size of first dispersers between good quality and poor quality corridors were tested for significance by Student’s *t* test separately within each corridor length × width treatment.
3. We explored how the experimental treatments affected the net movement and the rate of change in population size in the colonised patches. We define the rate of the population size change as the maximum rate of increase of the populations in the colonised patches over the first 10 days of the experiment, and the net movement of individuals as the number of individuals in the colonised patches predicted by the asymptotic model (see below). We focus on the initial 10 day period as Collembola eggs take 7–10 days to hatch (Fountain and Hopkin 2005), and eggs and very small individuals cannot be counted using our protocol. Therefore, we are able to attribute any change in the population size in the colonised patches over the first 10 days as being only due to dispersal from the source patches. Because we cannot mark individuals, we could not account for the dispersal of individuals from the colonised patches back to the source patches, and thus our measurement of the population sizes at the end of the 10-day period represents the net movement of individuals into the colonised patches during this time. While a limitation, our experiments nonetheless provide data on how the rate of colonisation of new patches depends on the features of the corridors connecting those patches, and thus how rapidly individuals can move through habitats. We used the numbers of individuals in the colonised patches over time to compare the different experimental treatments by fitting a non-linear mixed-effects model, with asymptote regression curves SSasympOrig fitted using ‘nlme’ package in R (R CoreTeam 2019). This approach was taken due to the non-linearity of the response (number of individuals in the colonised patches through time), and the fact that the data were inherently asymptotic. This approach firstly fits a basic model (in our case numbers of individuals ~ time) to the data which is allowed to vary amongst microcosms, then modifies the parameters of the basic model based on the specified predictor variables (in our case the experimental treatments) to assess whether there are any significant differences in these parameters between treatments (e.g. long vs short). Thus, any significance found in this model represents a significant difference in the slope or asymptote of the model between experimental treatments. Count data were log (*x* + 1) transformed and fixed effects in the model were corridor length, width, quality, and their two-way interactions, whilst the microcosm identity was included as a random effect to account for the variation in starting densities in the source patches among microcosms. Thus, the rate of change in population size was then defined as the maximum slope of this regression with a time component, whilst the net movement of individuals was the asymptotic value as calculated by the same model.

All statistical analysis was conducted in R (R CoreTeam 2019). Mean values are presented with 95% confidence intervals.

## Results

### The effects of corridor length, width and quality on the probability of an individual dispersing

**Fig. 1:**
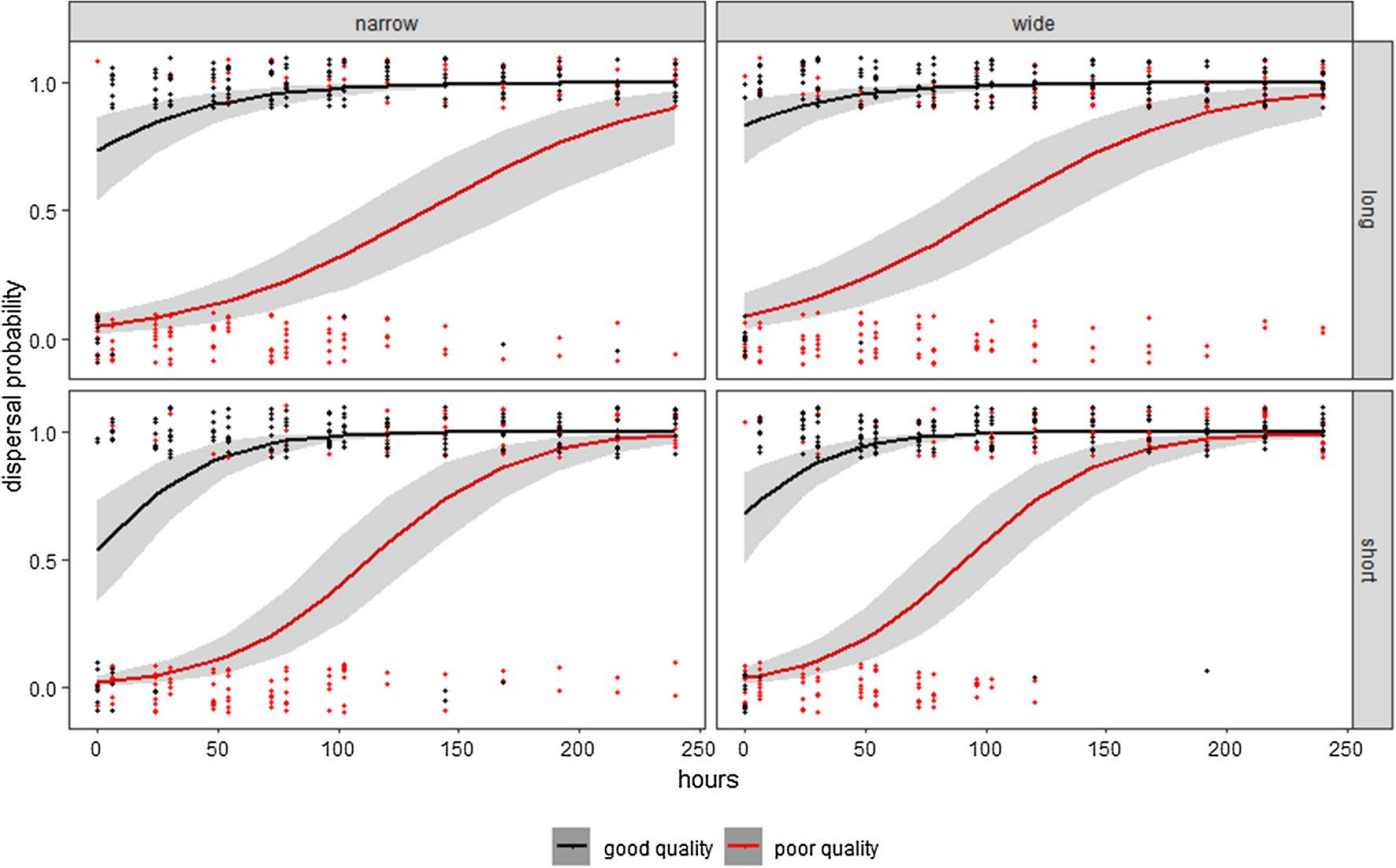
The effects of experimental treatments on the probability of an individual dispersing. Datapoints show the binary distributions (0 or 1) of dispersal events occurred in each replicate; *N* = 10 for each treatment. The black lines represent good quality corridors treatments; the red lines represent the poor quality corridor treatments. The areas highlighted in grey are 95% CI

Both corridor quality and time had positive effects on the probability of an individual dispersing (Table 1, Fig. 1). Corridor length had a negative effect on the probability of dispersal, whilst the width did not influence the probability of dispersal (Table 1). Corridor length, however, had a significant positive interaction with the time taken for dispersal to occur (Table 1), indicating that individuals took longer to disperse in longer corridors (Fig. 1). There were no significant effects of interactions between time and the other experimental treatments (Table 1).

**Table 1:** The effects of corridor length, width, and quality on the probability of dispersing

	Estimate	Std. error	*Z* value	*P* value	
(Intercept)	− 2.900081	0.441499	− 6.569	< 0.001	***
Time	0.021368	0.003035	7.041	< 0.001	***
Quality	3.917046	0.471131	8.314	< 0.001	***
Length	− 0.864439	0.441115	− 1.960	0.050	a
Width	0.603078	0.434320	1.389	0.165	
Hours × length	0.012095	0.003854	3.138	0.002	**
Hours × width	0.001179	0.003637	0.324	0.746	
Hours × quality	0.006573	0.005665	1.160	0.246	

Significance: 0 ‘***’, 0.001 ‘**’, 0.01 ‘*’, 0.05 ‘a’, 0.1 ‘’ 1

### The effects of corridor length, width and quality on the body size of first dispersers

**Fig. 2:**
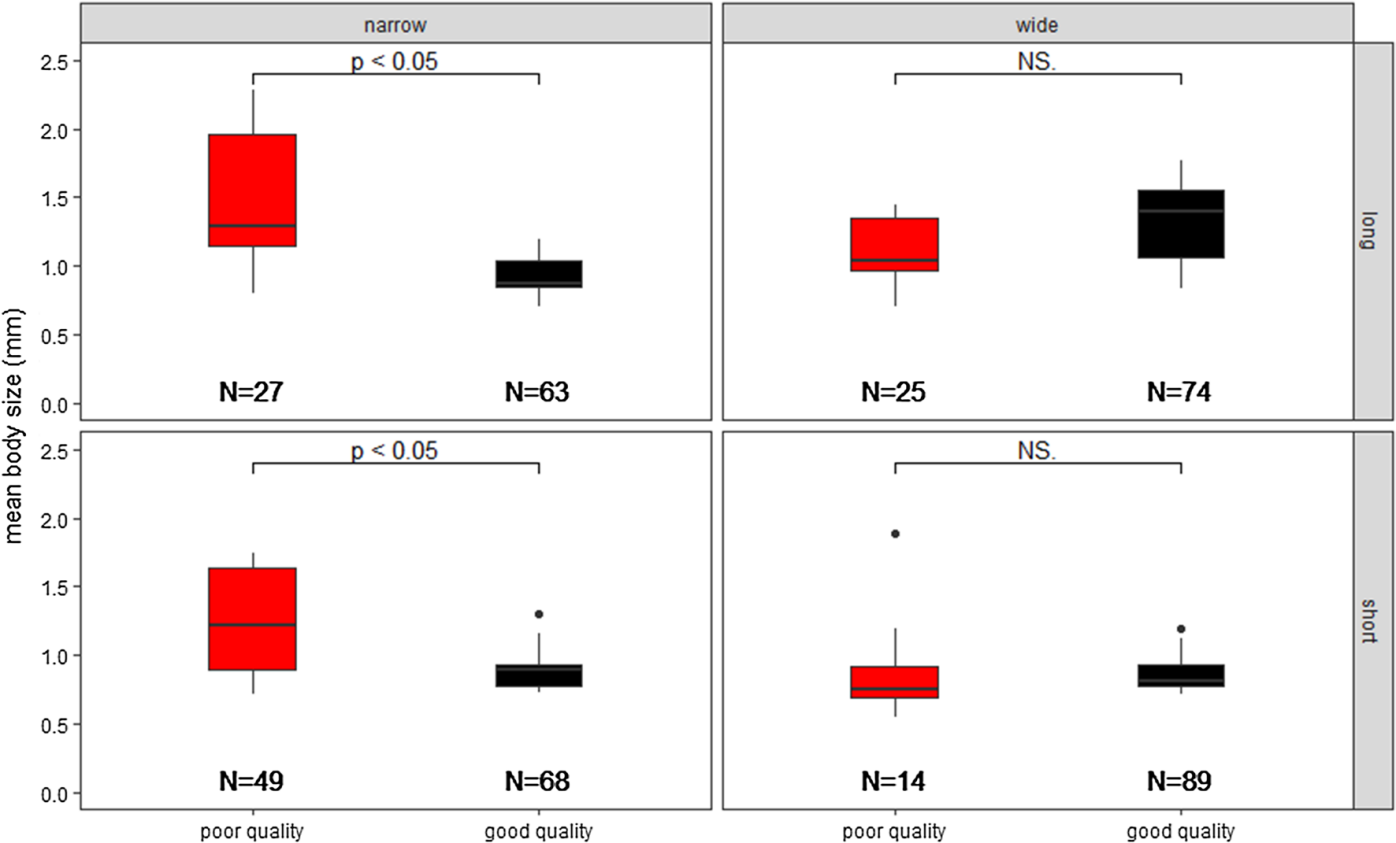
The effects of corridor length, width, and quality on the mean body size (mm) of first dispersers. *N* represents how many individuals were analysed for each treatment. The ends of the whiskers represent the maximum and the minimum limits, the ends of the boxes show the first and the third quartiles of the responses and the lines within the boxes represent the median; *P* < 0.05 denotes the significant differences between good quality (black filled) and poor quality (red filled) treatments

Corridor quality significantly affected the body size of first dispersers (Table 2), with larger individuals better able to disperse through poor quality corridors (Supp. Inf.). Corridor length and width had no effect on the body size of first dispersers (Table 2), however, there was a significant interaction between corridor width and quality (Table 2), as the body size of first dispersers in narrow and poor corridor treatments were significantly larger than those in narrow and good corridors (narrow × long with poor quality vs. narrow × long with good quality: *t* = 2.878, *P* = 0.01; narrow × short with poor quality vs. narrow × short with good quality: *t* = 2.276, *P* = 0.04, respectively). No significant corridor quality effects were found in wide corridor treatments (all *P* > 0.05, Fig. 2).

**Table 2:** The effects of corridor length, width, and quality on body size of first dispersers

	Estimate	Std. error	*Z* value	*P* value	
(Intercept)	1.38090	0.13114	10.530	< 0.001	***
Quality	− 0.43817	0.18545	− 2.363	0.018	*
Length	0.11073	0.18545	0.597	0.551	
Width	− 0.24042	0.18545	− 1.296	0.195	
Quality × length	− 0.06328	0.26227	− 0.241	0.809	
Length × width	− 0.23148	0.26227	− 0.883	0.378	
Quality × width	0.62234	0.26227	2.373	0.018	**
Quality × length × width	− 0.19279	0.37091	− 0.520	0.603	

Significance: 0 ‘***’, 0.001 ‘**’, 0.01 ‘*’, 0.05 ‘.’, 0.1 ‘’ 1

### The effects of corridor length, width, and quality on net movement and the rate of population size change in the colonised patches

**Fig. 3:**
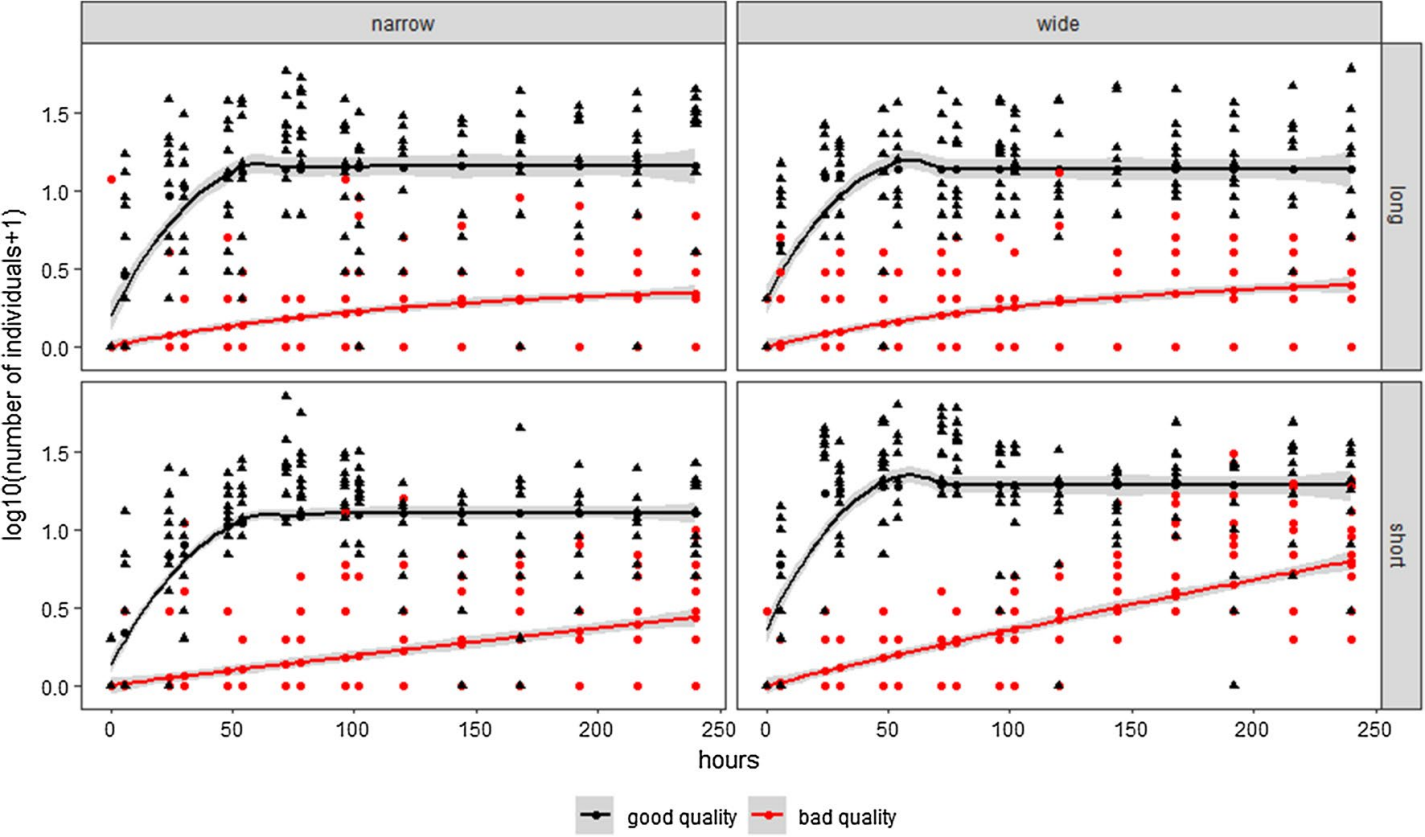
The effects of corridor length, width, and quality on the net movement and the rate of change in population size. Count data is log (*x* + 1) transformed; good quality corridor counts in each replicate are shown as black triangles, poor quality corridor counts in each replicate are shown as red circles; *N* = 10 for each treatment. The black lines represent good quality corridors treatments; the red lines represent the poor quality corridor treatments. The areas highlighted in grey are 95% CI

Significantly more individuals dispersed to the colonised patches over the 10-day initial period when corridor quality was good (Table 3, Fig. 3), and did so at a faster rate—with population size rapidly increasing in the colonised patches when corridor quality was good (Table 3, Fig. 3). Corridor length had a negative effect on the net movement of individuals, with more individuals moving through shorter corridors, whilst it had no effect on the rate of change in population size in the colonised patches (Table 3, Fig. 3). Corridor width had no effect on the net movement of individuals, however, it affected the rate of change in population size (Table 3, Fig. 3), with wider corridors facilitating a higher rate of change in population size. There were significant interactions between the corridor length and quality, corridor width and quality, and corridor length and width on the net movement of individuals (Table 3, Fig. 3). No significant interactions were found between the corridor length and quality, corridor width and quality, and corridor length and width on the rate of change in population size in the colonised patches (Table 3).

**Table 3:** The effects of corridor length, width, and quality on net movement and the rate of population size change in the colonised patches

Variables	Numerator *df*	Denominator *df*	*F* value	*P* value	
Net movement					
Intercept	1	1187	425.090	< 0.001	***
Length	1	1187	63.549	< 0.001	***
Quality	1	1187	1144.901	< 0.001	***
Width	1	1187	0.414	0.520	
Population size change rates					
Length: Quality	1	1187	3133.846	< 0.001	***
Quality: Width	1	1187	7.570	0.006	**
Length: Width	1	1187	6.457	0.011	*
Intercept	1	1187	651.813	< 0.001	***
Length	1	1187	1.971	0.161	
Quality	1	1187	52.204	< 0.001	***
Width	1	1187	14.199	0.002	**
Length: Quality	1	1187	3.013	0.083	a
Quality: Width	1	1187	0.887	0.347	
Length: Width	1	1187	2.505	0.114	

Significance: 0 ‘***’, 0.001 ‘**’, 0.01 ‘*’, 0.05 ‘a’, 0.1 ‘’ 1

## Discussion

Our results suggest that corridor length, width, and quality can play different roles in determining when and how often individuals disperse, with concomitant effects on the rate of change in population size of connected patches. Moreover, the attributes of corridors can determine which individuals disperse, leading to shifts in the body size of populations in the colonised patches. Overall, better quality corridors increased the probability of dispersal and net movement of individuals and did not act as a barrier to the dispersal of smaller individuals. Corridor length and width had no significant effect on the probability of individual dispersing, but length became more important when interacting with time. Corridor length affected the net movement of individuals but not the rate of change in population size, whilst corridor width affected the rate of change in population size but not the net movement of individuals.

We have demonstrated that the quality of corridors has an important effect on the probability of dispersal and net movement in our study system, a finding which supports previous theoretical prediction showing that corridor quality has a positive effect on the size of a metapopulation (Henein and Merriam 1990). Although the length and width of corridors have received much attention from ecologists (Andreassen et al. 1996; Haddad 2000; Beier and Gregory 2012), determining the optimal length and width for corridor construction remains difficult. For example, the best corridor length and width to increase movement can vary depending on the habitat preference of species and can be landscape-specific (Pérez-Hernández et al. 2015; Blazquez-Cabrera et al. 2016). Our results suggest that rather than focussing on the dimensions of corridors, investing effort in improving the quality of those corridors may prove the most effective way of ensuring species movement through fragmented landscapes.

Measuring the quality of corridors is challenging in the real-world system, and indeed what constitutes a good quality corridor is likely to vary between species. The sensitivity of a species to its habitat may in a large part drive perceived corridor quality (Lees and Peres 2008). In fact, corridors are often created with poorer-quality habitats (Haddad and Tewksbury 2005), which can result in a relatively rare use of corridors particularly for some habitat sensitive species. Given this, our results suggest that focussing on improving the quality of corridors is likely to increase the effectiveness of corridors. Exactly what constitutes an improvement will vary between species but might include, for example, increased three-dimensional structure for species which require cover to avoid predation, places to safely rest for species which cannot pass through the entire length of a corridor in a single dispersal event, the presence of food for species which disperse very slowly, or the removal of gaps in linear features followed by species such as bats (Bright 1998).

Not only do the quality of corridors affect the movement of individuals, also influence which individuals disperse. Our results suggest that the larger individuals were more able to travel through poor quality corridors, whilst good quality corridors allowed both large and small individuals to disperse. This body size effect is likely to be driven by the fact that larger individuals not only move faster through poor quality habitats but also have a lower surface-area to volume ratio, meaning that they desiccate more slowly. Thus, large individuals are more likely to successfully disperse through poor quality corridors which are low in humidity. Previous studies have shown that the dispersal ability of Collembolan species increases with body size (Johnson and Wellington 1983; Ulrich and Fiera 2010; Widenfalk et al. 2018), and thus this model system appears a good analogy of many natural systems, where body size is often shown to be a key determinant of dispersal ability (Hoekstra and Fagan 1998; Shurin et al. 2009; Forsman et al. 2011). Differences in dispersal ability based on body size has the potential to change the size structure of populations, in both the source and colonised patches. For example, significant sex-biased dispersal patterns to fragmented habitats were found in some saproxylic beetle species, leading to changes in the structure of populations (Bouget et al. 2015). Whilst the picture is not always clear-cut (e.g. Darcy and Eggleston 2005), such changes in the size distribution of populations have been shown to influence the resilience of populations and thus their probability of persisting into the future (Clements and Ozgul 2018). For example, using a small-scale experimental system, Clements and Ozgul (2016) has shown that a decline in mean body size of *Didinium nasutum* population occurred prior to their extinction.

The length and width of corridors also have important roles in dispersal processes and the rate of change in population size after colonisation, a phenomenon highlighted in previous studies (e.g. Baur and Baur 1992; Haddad 1999). However, we found contrasting effects of corridor length and width on the dispersal of individuals, with corridor length significantly effecting the net movement of individuals but not the rate at which this movement occurred, whilst corridor width significantly affected the rate at which individuals dispersed but not the net number of individuals which moved (Fig. 3). This demonstrates that the physical properties of corridors can differentially affect the dispersal of individuals, with concomitant effects on the abundance of populations in colonised patches. Such results support previous findings which have shown that the cost of dispersal increases with distance (Rousset and Gandon 2002; Bonte et al. 2012) and that wider corridors have lower edge-to-area ratios (Soule and Gilpin 1991; Haddad and Tewksbury 2005), which could enable higher dispersal rates. Our results go beyond these previous findings by showing that length, width, and quality can interact to alter the dispersal of individuals, primarily affecting the net movement of individuals into neighbouring patches.

Both the population and body size changes have important implications for the population growth after colonisation in our model system. We found higher rates of change in population size in colonisation patches connected with good quality and wide corridors, indicating higher population growth rates could be found in those patches. Moreover, as the body size of Collembola species are related to fecundity and mortality (Mallard et al. 2015), the changes in body size could possibly result in different population growth rates after colonisation. While we cannot measure the exact population growth in our model system, our results nonetheless provide the information on how corridors could have population-level effect on newly colonised populations.

In summary, we present the first comparison of the differential effects of corridor width, length, and quality on the probability of dispersal, net movement of individuals, and how these abiotic factors can shape the size, structure and the rate of change in population size after colonisation. Although low-quality corridors can still benefit conservation by directing species dispersal (Haddad and Tewksbury 2005), we suggest that the effectiveness of corridors could be increased by improving their quality. Future studies should consider the effect of corridor quality on a larger scale, with the potential to directly inform practical strategies for wildlife conservation in the field.

## Supplementary Information

Below is the link to the electronic supplementary material.

Supplementary file1 (DOCX 34 KB)

## Data Availability

Data available via the Dryad Digital Repository (10.5061/dryad.s4mw6m95r).
