# Supplementary material for: Corridor quality affects net movement, size of dispersers, and population growth in experimental microcosms
Source: Oecologia. 2021 Jan 9;195(2):547–56. doi: 10.1007/s00442-020-04834-2 (PMC7882584; doi:10.1007/s00442-020-04834-2)
Supplement: Supplementary file 1 — Supplementary file1 (DOCX 34 KB) [file 442_2020_4834_MOESM1_ESM.docx]

**Corridor quality affects net movement, size of dispersers and population growth in experimental microcosms**

Li, D^[[1]](#footnote-1)^, Clements, C, Shan, I.L.G and Memmott, J.

School of Biological Sciences,

Life Sciences Building

University of Bristol
24 Tyndall Avenue
Bristol, BS8 1TQ

**Supplementary materials:**

Effect of corridor length, width, and quality on the body size of first dispersers and non-dispersers

In our results we found that corridor quality affected the body size of dispersers in narrow corridors. This could be the result of 1) a bias towards larger individuals dispersing in poor quality corridor treatments, or 2) a bias towards small individuals dispersed in good quality corridors. In order to address this issue, we conducted an extra analysis on the comparison of first dispersers (colonisation patches) and non-dispersers (source patches) on the same treatment. The mean body size of dispersers and non-dispersers in same treatment was compared by a paired-*t*-test, and the results were shown as follows.

There were significant differences of body size of residents in long × wide × good treatment, long × narrow × poor treatment, and short × narrow × poor treatment (Table S1). The body sizes of dispersers in colonisation patches connected with narrow, poor quality corridors were significantly larger than those in source patches (Table S1, Fig. S1b and Fig. S1d). There were no significant differences between dispersers and non-dispersers in wide, poor quality corridor treatments ((Table S1, Fig. S1a and Fig. S1c). The body size of dispersers in colonisation patches connected with long, wide, and good quality corridors is significantly larger than those in source patches ((Table S1, Fig. S1e), whilst no significant differences of body size between dispersers and non-dispersers were found in other good quality corridor treatments ((Table S1, Fig. S1f, Fig. S1g, Fig. S1h).

The results support the idea that larger individuals are more likely to disperse in narrow and poor quality corridors.

**Table S1**. The comparison of body size between first dispersers and non-dispersers

| Corridor treatment | t | *p* |  |
| --- | --- | --- | --- |
| Long × wide × poor | 1.226 | 0.251 |  |
| Long × wide × good | 2.270 | 0.049 | * |
| Long × narrow × poor | 2.303 | 0.047 | * |
| Long × narrow × good | 0.461 | 0.656 |  |
| Short × wide × poor | 0.543 | 0.600 |  |
| Short × wide × good | 1.209 | 0.257 |  |
| Short × narrow × poor | 2.297 | 0.047 | * |
| Short × narrow × good | 1.394 | 0.197 |  |

Significance: 0 ‘***’ 0.001 ‘**’ 0.01 ‘*’ 0.05 ‘.’ 0.1 ‘ ’ 1


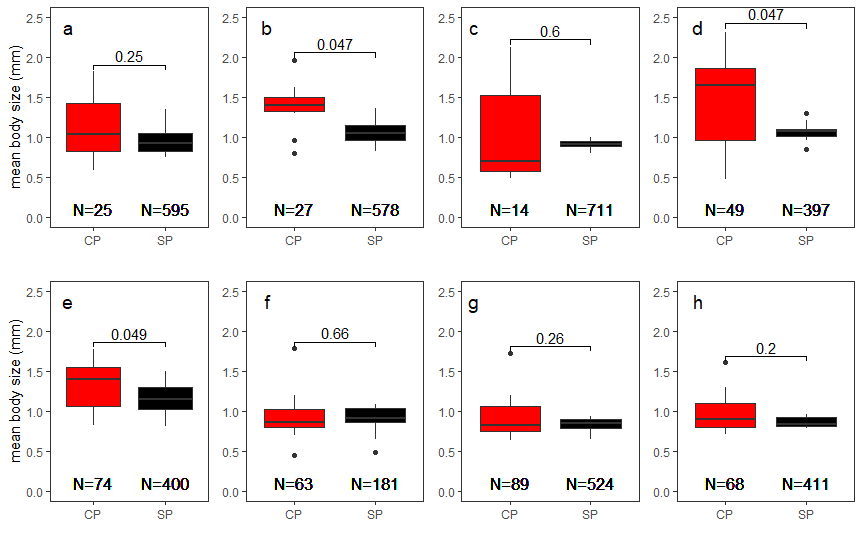


**Figure S1**. The comparison of mean body size of residents in colonisation patches (CP) and source patches (SP). a = long × wide × poor quality corridor treatment; b = long × narrow × poor quality corridor treatment; c = short × wide × poor quality corridor treatment; d = short × narrow × poor quality corridor treatment; e = long × wide × good quality corridor treatment; f = long × narrow × good quality corridor treatment; g = short × wide × good quality corridor treatment; h = short × narrow × good quality corridor treatment.

1. Correspondence author: [Dongbo.Li@bristol.ac.uk](mailto:Dongbo.Li@bristol.ac.uk) [↑](#footnote-ref-1)
